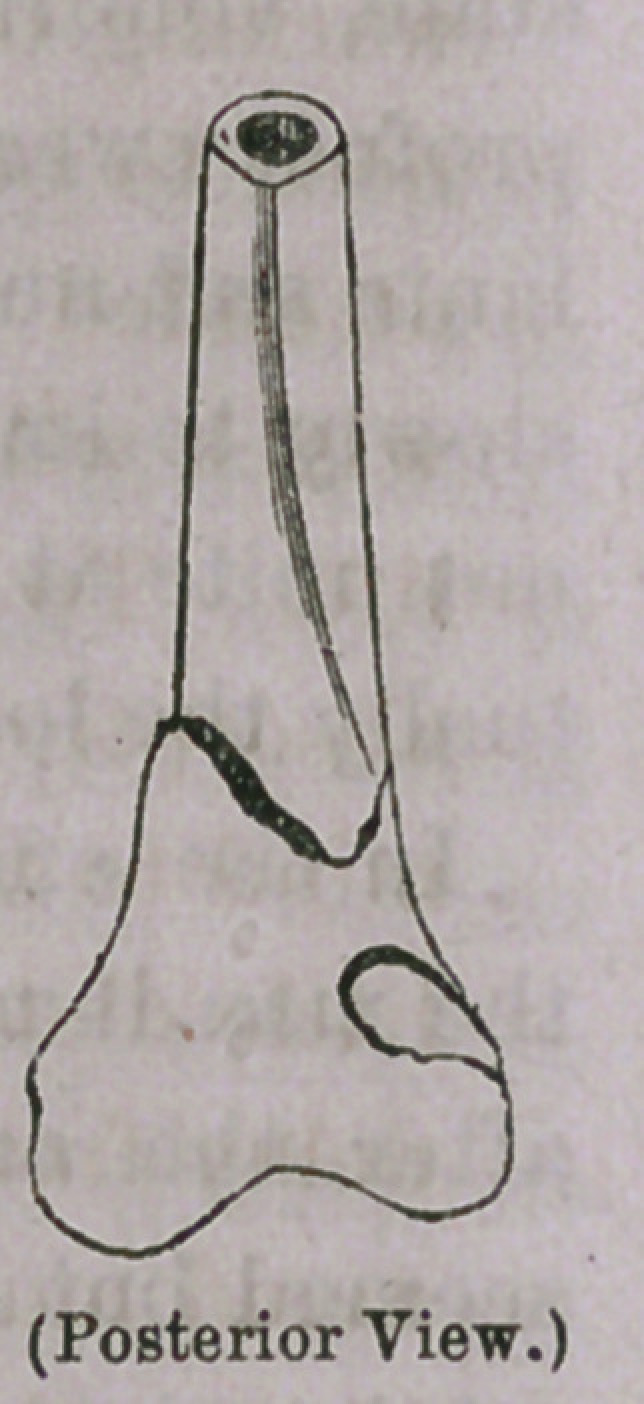# A Case of Injury to the Knee-Joint

**Published:** 1865-07

**Authors:** J. R. Lothrop


ART. II.—*A Case of Injury to the Knee-Joint.* By J. R. LOTHROP, M. D.

In this case the injury was caused by the passage of the flange of a car-wheel over the knee. It occurred in the following manner: The man injured was about to uncouple some cars while the train (one made up of loaded freight cars) was slowly moving. As he stepped in between the cars, his foot caught between two rails—this happening near a switch where the rails were converging. Being unable to extricate his foot, he was thrown down under the cars upon his right side, his head in the direction of the moving train, his foot bent over the inner rail on which the wheels were running, and his right knee brought up by the side of, but not upon, the same rail. It appears that the right limb flexed lay upon a cross-tie, the outer side resting on the tie. The head of the spike which fastens the rail pressed upon the outer side of the femur and near the joint. In this position the knee was pushed close to the rail, so that the flange of the wheel as it passed along cut into the joint on its inner side, striking heavily the inner condyle of the femur, and bruising off the articular cartilage, some of which appeared in the wound. At first it was supposed the knee had taken but little of the weight of the car, and that the injury inflicted was limited to the opening of the joint; but it was afterwards found that the weight had been sufficient to fracture the femur a short distance above the joint, though, as no displacement occurred, it was not suspected at the time. It seems very probable, both from the position in which the man lay, and the appearance of the boot which he wore, (a very heavy and thick-soled one), that the wheels passed over the foot as it lay bent over the rail. The marks left on the boot indicated that it had been subjected to violence. But whether this is so or not, it is sufficient to say that the foot escaped with slight injury, a fact somewhat surprising if it was subjected to the weight of a loaded freight car.

The man was carried to the Buffalo General Hospital not many hours after the accident, and was soon seen by me. There was then a wound on the inner side of the knee-joint, about four inches in length, extending around to the front somewhat. That it penetrated the joint was evident from the escape of the synovial fluid and from pieces of articular cartilage in the wound. On the outer side was also a wound deep and defined, corresponding to the head of the spike. After a careful examination, finding no evidence of a fracture, and supposing, from the appearance of the wound, that it was of the nature of an incised wound, it was determined to make the attempt to save the limb. I think from the appearance of the knee there was no ground for suspecting that it had been subjected to the violent pressure which after events proved to have been inflicted. The motions of the joint were made, but there was no crepitus or mobility near the joint to raise the surmise of a fracture.

Thinking it best to attempt to save the limb, it was kept in a slightly flexed position, and cold water applied. For some time everything went on well. The patient's condition was good; his appetite and strength kept up, and there was not much pain or constitutional disturbance. But after a time pus formed, and though the wound seemed sufficient to afford it free exit, it burrowed about the joint. Openings were made, but the formation continued, and the patient's strength began to fail. Still, as too often happens in such cases, amputation was delayed in the hope that a change might take place for the better; until the condition of the patient and the joint was such that nothing remained but to remove it. Just one month had passed since the injury, and the patient's state, under great suppuration, irritative fever and diarrhœa which had set in, rendered it doubtful if he would survive amputation. It had come to this, an experience more common than perhaps it ought to be, that in the attempt to save a limb, both limb and life are put in jeopardy. However, there being no other procedure left, unpromising as affairs looked, the limb was removed. The combined effects of ether and the operation, left the patient in a state of prostration from which he seemed little likely to rally. For about two weeks he was in a very critical state, vomiting some, with a constant diarrhœa, small quick pulse, profuse sweating and pinched features. By as free use of stimulants and nutritious diet as could be borne, after a time he began slowly to improve, and after a long time recovered. The flaps did not unite at first, though but little sloughing took place, and eventually the lower end of the bone separated by necrosis.

In the end, after several months, a good stump resulted, and the man is now as well as ever he was before the accident. Soon after the amputation, when his condition was worse, and there seemed but little hope of his improving, it was determined to administer strychnine in small doses. Accordingly, a twentieth of a grain was given every fourth hour. A marked change for the better soon took place. The diarrhœa ceased, the appetite improved, and the face appeared brighter. The strychnine was continued until it caused twitching of the limbs and then suspended. After its disuse the diarrhœa reappeared, and the patient seemed somewhat worse. These symptoms ceased upon the renewal of the remedy. This is mentioned, not that it may be set down as an instance of a certain beneficial operation of strychnine as a stimulant in low conditions, but only that it may be inferred that in some way a good result following its use seemed quite probable.

In the removed limb the cavity of the joint was found filled with purulent matter, the cartilages softened, disorganized, and at the point where the condyle was struck by the edge of the wheel, entirely gone. Pus was found burrowing deeply both above and below the knee, and as high up the thigh as the line of fracture extended. Slight attempts at repair were evident at some points of the fracture; osseous material being found in small quantities about the break.

An examination of the bone after removal revealed the existence of a fracture without displacement just above the joint, a split at the line of union of the epiphysis on the inner and front aspect of the bone, and a depression in the bone on the outer and posterior side corresponding in size to the head of the spike on which it bore. The crushing force exerted upon the bone was therefore great. It is quite remarkable that such an extensive fracture of bone could occur and yet no displacement follow. The accompanying representation will give some idea of the nature of the injury to the bone.

(Front View.)

(Posterior View.)

This case in some respects resembles injuries of the knee-joint occurring in military practice, in which the force causing the wound is great, the shock severe, and the injury to the bones entering into the joint extensive. Such cases generally do not admit of attempts to conserve, and in such cases secondary amputation is almost invariably fatal. That recovery took place does not invalidate the rule that primary amputation is safest and wisest. It is not, therefore, intended to bring this forward as in any measure establishing the correctness of the practice. It would have been far better to have removed the limb very soon after the injury was received. Had the amount of injury to the bone been fully understood, probably no delay would have been suffered. But some writers state that the knee joint may be opened and recovery will follow; and even that recovery may follow when suppuration has taken place, if the openings are made sufficiently large to give free escape to the pus. Without doubt recoveries have been observed, and limbs saved, but in many cases reported, there is probably a little doubt whether the joint has been opened. In gun-shot wounds, for instance, a ball may pass near without opening the cavity of the joint, and great swelling and suppuration ensue, ending, however, in recovery. Such cases are not to be classed with or furnish precedents for those in which the joint is actually opened, air allowed to enter, and long suppuration follows, with the familiar, severe symptoms of joint injury, especially in one as important as the knee. Sharp cutting instruments may enter a joint inflicting no injury upon the bone, and by careful treatment, such as closing the wound, not much trouble follow. Such cases are seen in civil practice, and the fact that they are saved goes some way to influence the judgment in severer cases in which delay is mischievous, and early amputation the only proper treatment.

Injuries to the knee often deceive us into a fatal delay, even when the bones suffer much, by the absence of early severe symptoms. They often go on well for a time, and the condition of the patient remains good; and nothing but experience can give us warning of the dangers which will occur as the case goes on. We need to be prepared for the wearing and exhausting suppuration attended with abscesses burrowing about extensively, and diarrhœa which will come on at a later period to waste the strength and life. To these dangers may be added, the liability of purulent absorption. It certainly seems sometimes difficult to make up one's mind to the necessity of resorting to amputation when there is so little disturbance or pain, and especially when the patient begs for delay from an inability to comprehend the danger, and a desire to avoid mutilation. So that it happens that the period most favorable for the operation, and that perhaps in which it may be safely performed, passes by sooner than one is prepared for. The symptoms often become very serious in a short time.

An injury like the one related above, would, in all probability, be attended with breaking off of pieces of bone, which, acting like foreign bodies, would entirely preclude all idea of closing up the wound, and thereby favoring an early subsidence of the threatened mischief. In this respect it resembles gun-shot wounds of the knee joint, in which even if the bone is not splintered, the track of tha ball must suppurate, and therefore the wound must be kept open. This being the case some surgeons have practiced free incision into the joint and report recoveries. The cases reported by Dr. Moses, an advocate, in his earlier military experience of delay, are probably familiar to most readers, but they will not fail to notice that in his last report he speaks with much less confidence of the propriety of attempts to save the limb when the knee has met with severe injury.

The experience of our army surgeons when collected and published, will give us valuable and abundant instruction in the matter of severe knee joint injuries. Heretofore military surgeons have declared early removal of the limb the only safe and proper treatment. The experience of many, of most we may say, has been that unless this is done a fatal result will in all cases follow. McLeod, in his Notes on the Surgery of the Crimean War, says: "I have never met with one instance of recovery in which the joint was distinctly opened, and the bones forming it much injured by a ball, unless the limb was removed."

This case of recovery after secondary amputation, is to be set down as one of the exceptional cases which sometimes occur. It will be apparent to most what reasons influenced to delay, viz: the early absence of severe symptoms, the desire to save a limb, the partial resemblance of the wound to an incised wound, the obscurity of the bone injuries, the desire of the patient to avoid, if possible, mutilation, and I may add what perhaps I should not, had the result been different, a failure to recognize the absolute but deplorable necessity which experience has led others to appreciate, viz: an early resort to amputation. The result, therefore, does not justify the treatment, and this example may serve as a useful, warning, bnt by no means as a guide in a case of similar injury.

## Figures and Tables

**Figure f1:**
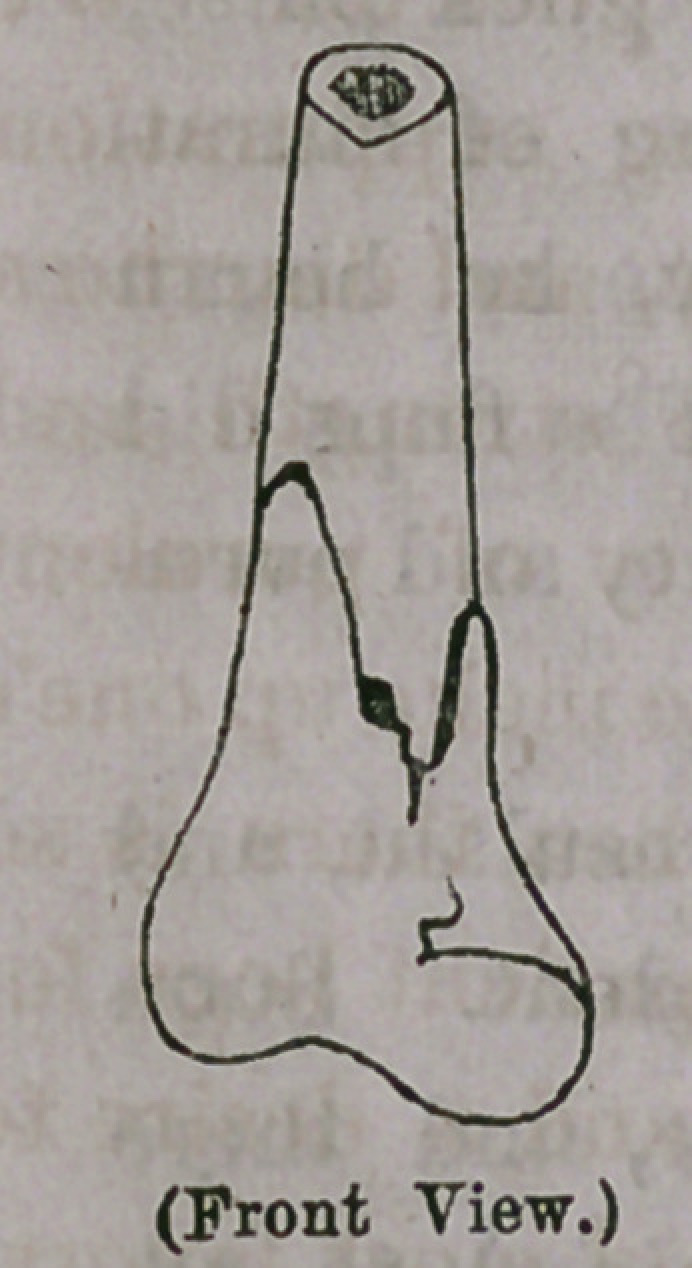


**Figure f2:**